# *TTN* as a candidate gene for distal arthrogryposis type 10 pathogenesis

**DOI:** 10.1186/s43141-022-00405-5

**Published:** 2022-08-11

**Authors:** Anik Biswas, Sudipta Deb Nath, Tamim Ahsan, M. Monir Hossain, Sharif Akhteruzzaman, Abu Ashfaqur Sajib

**Affiliations:** 1 Department of Genetic Engineering & Biotechnology, University of Dhaka, Dhaka, 1000 Bangladesh; 2 Molecular Biotechnology Division, National Institute of Biotechnology, Savar, Dhaka, 1349 Bangladesh; 3 Department of Neonatal Medicine, Bangladesh Institute of Child Health, Dhaka, 1207 Bangladesh

**Keywords:** Distal arthrogryposis, Congenital contractures, Protein-protein interaction network, Titin, TTN

## Abstract

**Background:**

Arthrogryposis is a medical term used to describe congenital contractures which often affect multiple limbs. Distal arthrogryposis (DA) is one of the major categories of arthrogryposis that primarily affects the distal parts of the body, i.e., the hands and the legs. Although ten different types and several subtypes of DAs have been described, the genes associated with each of these DAs are yet to be characterized. Distal arthrogryposis type 10 (DA10) is a rare genetic disease, which is distinguished from the other arthrogryposis types by plantar flexion contractures resulting in toe-walking during infancy as well as variability in contractures of the hip, hamstring, elbow, wrist and finger joints with no ocular or neurological abnormalities. Symptoms of DA10 indicate impairment specifically in the musculoskeletal system. DA10 is still poorly studied.

**Aim:**

The objective of this study was to identify the candidate gene for DA10 by scrutinizing the protein-protein interaction (PPI) networks using in silico tools.

**Results:**

Among the genes that reside within the previously reported genomic coordinates (human chromosome assembly 38 or GRCh38 coordinates 2:179,700,000–188,500,000) of the causative agent of DA10, only *TTN* (the gene that codes for the protein Titin or TTN) follows the expression pattern similar to the other known DA associated genes and its expression is predominant in the skeletal and heart muscles. Titin also participates in biological pathways and processes relevant to arthrogryposes. TTN-related known skeletal muscle disorders follow the autosomal-dominant pattern of inheritance, which is a common characteristic of distal arthrogryposes as well.

**Conclusion:**

Based on the findings of the analyses and their correlation with previous reports, *TTN* appears to be the candidate gene for DA10. Our attempt to discover a potential candidate gene may eventually lead to an understanding of disease mechanism and possible treatment strategies, as well as demonstrate the suitability of PPI in the search for candidate genes.

**Supplementary Information:**

The online version contains supplementary material available at 10.1186/s43141-022-00405-5.

## Background

“Arthrogryposis” is derived from the Greek words “arthron”, meaning joint, and “gryposis” meaning curvature [1]. Arthrogryposis and arthrogryposis multiplex congenita (AMC) are used as generalized terms to describe inborn congenital contractures, which often affect multiple limbs [2]. These descriptive terms, however, do not denote any specific diagnosis for such conditions as AMCs have been recognized in different conditions [2, 3]. One child in every 3000 to 5100 live births is born with arthrogryposis with different levels of penetrance [1, 4]. Decreased in utero fetal movement is observed in every affected case, but the cause of such diminished movement can be vastly different [2]. In addition to maternal effects such as a bicornuate uterus, oligohydramnios, or intrauterine crowding (observed in twins) [5], mutations in certain genes contribute to AMC development [6].

Bamshad et al. described the most widely accepted clinical classification of AMC. According to this classification, AMC can be caused by amyoplasia, neurological abnormalities, or distal arthrogryposis. Amyoplasia is caused by impaired muscle growth leading to contractures that affect every symmetrical joint of the body. Internally rotated shoulders, extended elbows, flexed wrists, dislocated hips, equinovarus contractures in the feet, rigid fingers, and thumbs are common symptoms in these cases [5]. Many patients also have a mid-facial hemangioma. Most of these patients, however, have normal intelligence. Hereditary components have not been established for AMC caused by amyoplasia, and surgical intervention is usually needed for treatment [1]. Complications during central nervous system development and peripheral neuropathies can also lead to decreased fetal movement and arthrogryposis [5]. Occasionally, auto-antibodies from the mother can target fetal acetylcholine receptors and cause neuropathies in the fetus [1]. AMCs are inherited in an autosomal recessive manner, whereas the types and sub-types of distal arthrogryposes (DAs) are mostly autosomal dominant in nature [1, 4, 5, 7–9]. DA primarily affects the distal parts of the body, i.e., the hands and the legs. It is the second largest cause of AMC after amyoplasia [10]. According to the classification proposed by Bamshad et al [5], DA is characterized by multiple congenital contractures without a primary muscular or neurological disorder. DA patients are usually treated with the goal of improving the motor function of affected joints, strengthening muscles with physiotherapies, and correcting deformities through surgery [11].

Ten different types and several subtypes of DAs have been characterized and classified according to their proportion of shared features [5]. However, the genes associated with each type of DAs are yet to be categorized. DA10 is distinguished from the AMCs and other DAs by plantar flexion contractures, resulting in toe-walking during infancy [12, 13]. Additionally, patients show variability in contractures of the hip, hamstring, elbow, wrist, and finger joints [13]. Like the other DAs, DA10 is still poorly studied. In this study, we scrutinized the interactions among the already known arthrogryposis-associated gene products through protein-protein interaction (PPI) network analyses as well as database search and explored the potential candidate gene associated with the development of distal arthrogryposis type 10 (DA10).

## Methods

### Identification of the interactors of arthrogryposis-associated proteins

The list of genes that are known to be associated with different types and subtypes of DA (Table 1) was retrieved from the Online Mendelian Inheritance in Man^®^ (OMIM^®^) database [6]*.* These genes were used as input in NetworkAnalyst 3.0 [14] to identify their interactors through exploring the non-redundant set of physical molecular interaction data at the IMEx [15] and the STRING (with experimental evidence and high confidence score) databases [16]. Based on these protein-protein interaction networks, the associated pathways and processes were identified (with a false discovery rate or FDR <0.05) from the Gene Ontology (Biological Process) [17], PANTHER (Biological Process) [18], Reactome [19] and KEGG [20] databases and roles of different interactors in these pathways and processes were retrieved. Based on their roles, the interactor proteins that are relevant to arthrogryposis were identified and their chromosomal locations were collected from database resources at the National Center for Biotechnology Information (NCBI) [21].

**Table 1:** List of genes associated with different arthrogryposes [6]

***Arthrogryposis type***	***Associated gene***	Mode of inheritance
Arthrogryposis multiplex congenita 1	*LGI4*	Autosomal recessive
Arthrogryposis multiplex congenita 2	*ERGIC1*	Autosomal recessive
Arthrogryposis multiplex congenita 3	*SYNE1*	Autosomal recessive
Arthrogryposis multiplex congenita 4	*SCYL2*	Autosomal recessive
Arthrogryposis multiplex congenita 5	*TOR1A*	Autosomal recessive
Distal arthrogryposis, type 1A	*TPM2*	Autosomal dominant
Distal arthrogryposis, type 1B	*MYBPC1*	Autosomal dominant
Distal arthrogryposis, type 1C	*MYLPF*	Autosomal dominant
Distal arthrogryposis, type 2A (Freeman-Sheldon syndrome)	*MYH3*	Autosomal dominant
Distal arthrogryposis, type 2B1	*TNNI2*	Autosomal dominant
Distal arthrogryposis, type 2B2	*TNNT3*	Autosomal dominant
Distal arthrogryposis, type 2B3 (Sheldon-Hall syndrome)	*MYH3*	Autosomal dominant
Distal arthrogryposis, type 2B4	*TPM2*	Autosomal dominant
Distal arthrogryposis, type 3	*PIEZO2*	Autosomal dominant
Distal arthrogryposis, type 4	Not Mapped	Autosomal dominant
Distal arthrogryposis, type 5	*PIEZO2*	Autosomal dominant
Distal arthrogryposis, type 5D	*ECEL1*	Autosomal dominant
Distal arthrogryposis, type 6	Not Mapped	Autosomal dominant
Distal arthrogryposis, type 7 (Trismus-pseudocamptodactyly syndrome)	*MYH8*	Autosomal dominant
Distal arthrogryposis, type 8 (Contractures, pterygia, and spondylocarpotarsal fusion syndrome 1A)	*MYH3*	Autosomal dominant
Distal arthrogryposis, type 9	*FBN2*	Autosomal dominant
Distal arthrogryposis, type 10	Cytogenetic location: 2q31.3–q32.1 Genomic coordinates (GRCh38): 2:179,700,000–188,500,000	Autosomal dominant

### Identification of functionally relevant genes associated with arthrogryposis

Based on genome-wide linkage analysis, a previous study reported that the candidate gene for DA10 resides on human chromosome 2 within the region between 179,700,000 and 188,500,000 bps in the chromosome assembly 38 (GRCh38) (equivalent to genomic coordinates 2:179,390,716–179,672,150 in the human chromosome assembly 37 or GRCh37) [12]. The list of genes that are located within these genomic coordinates on human chromosome 2 was retrieved from the Atlas of Genetics and Cytogenetics in Oncology and Haematology database [22]. In addition, the genes of the interactor proteins that are relevant to arthrogryposis (mentioned in the previous section), and reside within the previously suggested region [12] or very close to the defined region (as genetic linkage does not provide absolute distance in base pairs) were identified. Expression and functions of these gene-encoded proteins were explored in the Human Protein Atlas database [23] and genes functionally relevant to DA10 were identified.

### Identification of potential candidate gene

The functionally relevant genes to DA10 (identified through PPI network analysis as well as the Atlas of Genetics and Cytogenetics in Oncology and Haematology database) were used as input in NetworkAnalyst 3.0 [14] to identify their roles in biological pathways and processes (FDR <0.05) based on the data at the STRING database (with experimental evidence and high confidence score). Participation of these proteins in muscle contraction-related pathways was explored by identifying the pathways and processes (FDR <0.05) from the Gene Ontology (Biological Process), PANTHER (Biological Process), Reactome, and KEGG pathway databases, and their relevance to DA10 pathogenesis was scrutinized.

## Results

### Interactions among the known arthrogryposis-associated gene products

**Fig. 1:**
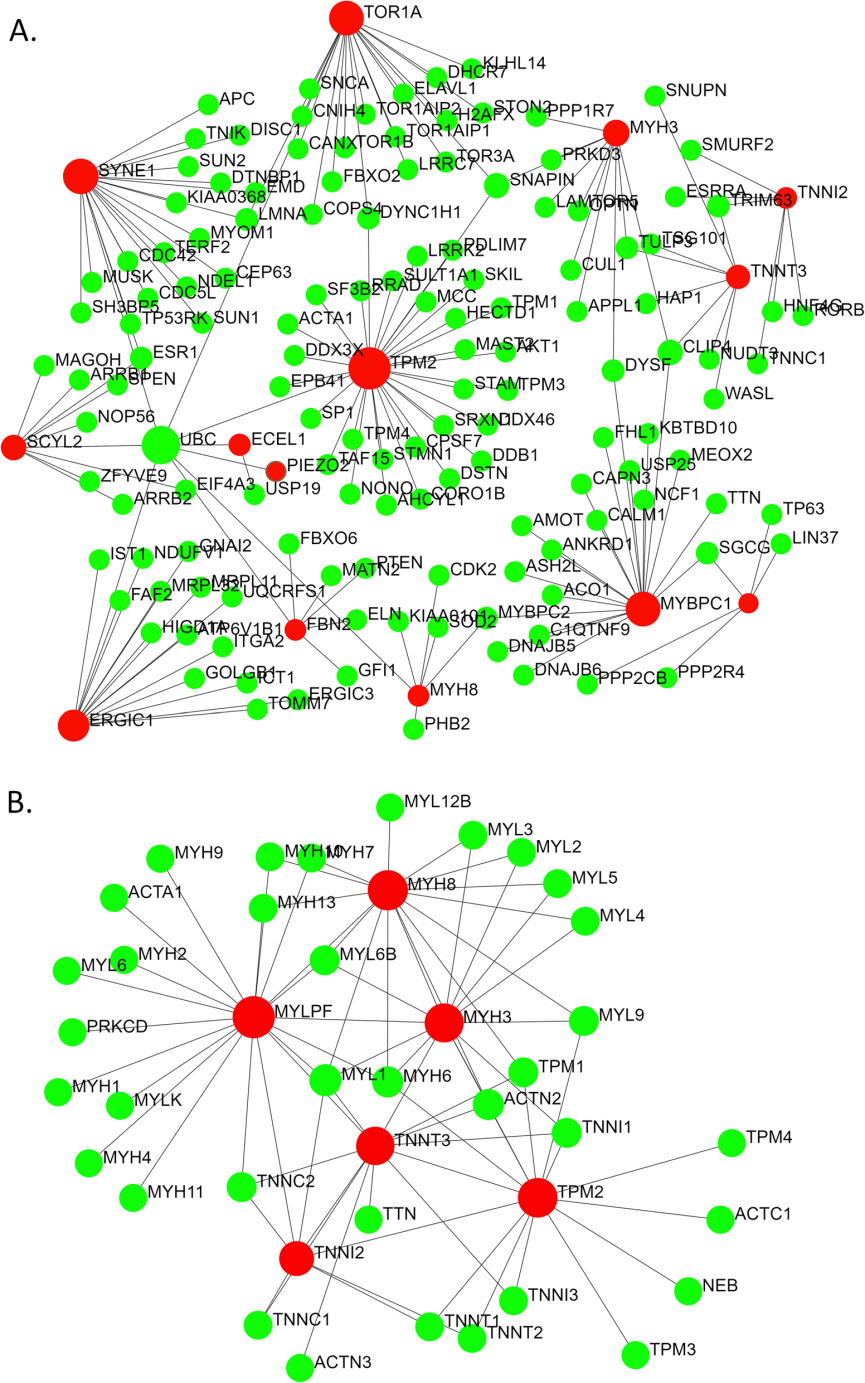
PPI networks generated using 15 known genes associated with AMCs and DAs based on (**A)** IMEx and (**B)** STRING databases. AMC and DA-associated gene-encoded proteins are shown in red and the other interacting proteins are shown in green

Mutations in different genes cause arthrogryposis (Table 1). Even mutations at different loci in certain genes are associated with several types of DAs (Table 1). While visualizing the interactions among the proteins encoded by the 15 arthrogryposes associated genes using the IMEx interactome database, a total of 151 nodes were found that incorporated 14 of the query genes as nodes (Fig. 1A). The biological pathways and processes (retrieved from the Gene Ontology, PANTHER, Reactome, and KEGG databases) associated with these 151 nodal proteins (Table 2) were similar.

**Table 2:** Pathways associated with the interacting proteins (identified through IMEx database)

GO:BP		PANTHER:BP		Reactome		KEGG	
Pathway	FDR	Pathway	FDR	Pathway	FDR	Pathway	FDR
Actin filament-based movement	1.37E−12	Muscle contraction	7.64E-14	Striated muscle contraction	2.27E−13	Hypertrophic cardiomyopathy	4.08E−06
Actin filament-based process	1.91E−09			Muscle contraction	4.01E−12	Dilated cardiomyopathy	4.08E−06
Cytoskeleton organization	2.11E−07			Smooth muscle contraction	0.00305	Adrenergic signaling in cardiomyopathy	0.00176
Cellular membrane organization	2.64E−06						
Muscle organ development	2.64E−06						
Striated muscle tissue development	1.69E−05						

*FDR* false discovery rate

The same query genes were used as input to search protein-protein interactions and associated biological processes in the STRING interactome database (Table 3). Among the five sub-networks obtained, only the largest sub-network showed relevance to AMCs and DAs. There was considerable overlap among the pathways and processes identified based on IMEx and STRING databases. The total number of distinct nodes (proteins) in these two PPI networks obtained through IMEx and STRING interactome databases summed up to 180. These genes and their corresponding number of interactions are shown in Supplementary Table 1.

**Table 3:** Pathways associated with the interacting proteins (identified through STRING database)

GO:BP		PANTHER:BP		Reactome		KEGG	
Pathway	FDR	Pathway	FDR	Pathway	FDR	Pathway	FDR
Actin filament-based movement	7.94E−53	Muscle contraction	2.01E−48	Muscle contraction	4.22E−54	Hypertrophic cardiomyopathy	1.66E−18
Actin filament-based process	1.84E−35			Striated muscle contraction	2.40E−43	Dilated cardiomyopathy	2.49E−18
Striated muscle contraction	2.02E−19			Smooth muscle contraction	1.23E−16	Cardiac muscle contraction	7.97E−16
Regulation of muscle contraction	8.79E−14					Adrenergic signaling in cardiomyopathy	2.6E−12
Striated muscle tissue development	1.20E−12						
Muscle organ development	6.15E−12						
Actin cytoskeleton organization	6.07E−10						
Muscle cell differentiation	1.79E−06						
Cytoskeleton organization	2.00E−06						

*FDR* false discovery rate

Since the known genes that are associated with different types of distal arthrogryposis appeared to be connected through PPI, we hypothesized the presence of the candidate gene for DA10 to be within the network. Genome-wide linkage analysis of 5 generations of a family with DA10 has pointed to the cytogenetic location 2q31.3–q32.1 to be associated with DA10. This location corresponds to the GRCh38 genomic coordinates 2:179,700,000–188,500,000, and GRCh37 genomic coordinates 2: 179,390,716–179,672,150 (Table 1) [6, 12]. Therefore, the member proteins of the PPI networks that reside on chromosome 2 were identified. Among the 180 nodes (proteins), only 8 are encoded by genes that reside on human chromosome 2 (Table 4). Only one of these genes, *TTN*, appears to be very closely located to the predicted genomic coordinates of the DA10 candidate gene (Table 1).

**Table 4:** List and genomic coordinates of the genes in the PPI network that are positioned on human chromosome 2

Gene	Chromosome	Genomic coordinates
		GRCh38
*CLIP4*	2	29,097,681...29,183,808
*PRKD3*	2	37,250,502...37,324,833
*ATP6V1B1*	2	70,935,900...70,965,431
*DYSF*	2	71,453,154...71,686,763
*KBTBD10*	2	169,509,702...169,526,258
***TTN***	2	178,525,989...178,807,423 (GRCh37: 179,390,716...179,672,150)
*ECEL1*	2	232,479,827...232,487,834
*PPP1R7*	2	241,149,573...241,183,652

*GRC* Genome Reference Consortium

**Fig. 2:**
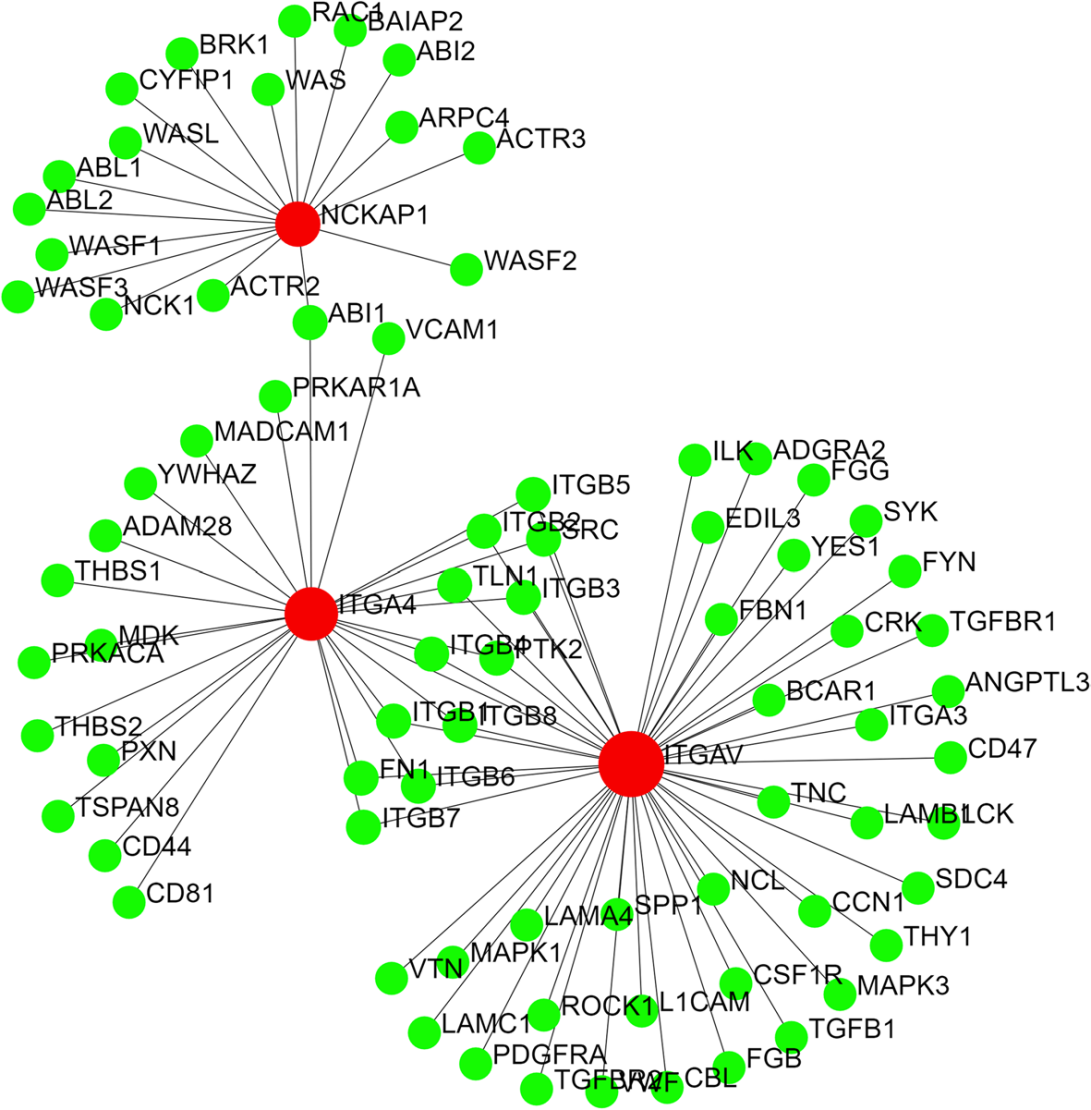
PPI sub-network that includes ITGAV, ITGA4, and NCKAP1

We also looked for other potential candidate genes for DA10 by retrieving the list of genes that reside within 2q31.3–q32.1 (Table 5). Thirty-three genes reside within this region on human chromosome 2. Among the 12 PPI sub-networks identified using these genes as input in Metaboanalyst, only one sub-network comprising ITGAV, NCKAP1, and ITGA4 appeared to be involved in the regulation of actin cytoskeleton (with experimental evidence and high confidence score), which is relevant to arthrogryposis development [24] (Fig. 2).

**Table 5:** List of the genes at 2q31.3–q32.1 on human chromosome 2

Gene name	GRCh38 location	Cytogenetic location	Gene product
*CWC22*	179944.877	2q31.3	CWC22 spliceosome-associated protein homolog
*SCHLAP1*	180692.104	2q31.3	SWI/SNF complex antagonist associated with prostate cancer 1
*UBE2E3*	180980.385	2q31.3	Ubiquitin-conjugating enzyme E2 E3
*LINC01934*	181123.837	2q31.3	Long intergenic non-protein coding RNA 1934
*MIR4437*	181305.593	2q31.3	MicroRNA 4437
*ITGA4*	181456.892	2q31.3	Integrin subunit alpha 4
*CERKL*	181536.674	2q31.3	Ceramide kinase like
*NEUROD1*	181676.106	2q31.3	Neuronal differentiation 1
*ITPRID2*	181891.730	2q31.3	ITPR interacting domain containing 2
*PPP1R1C*	181985.853	2q31.3–q32.1	Protein phosphatase 1 regulatory inhibitor subunit 1C
*PDE1A*	182140.041	2q32.1	Phosphodiesterase 1A
*DNAJC10*	182716.257	2q32.1	DnaJ heat shock protein family (Hsp40) member C10
*FRZB*	182833.276	2q32.1	Frizzled-related protein
*NCKAP1*	182909.115	2q32.1	NCK-associated protein 1
*DUSP19*	183078.747	2q32.1	Dual specificity phosphatase 19
*NUP35*	183124.355	2q32.1	Nucleoporin 35
*MIR548AE1*	184378.975	2q32.1	MicroRNA 548ae-1
*ZNF804A*	184598.366	2q32.1	Zinc finger protein 804A
*LOC105373782*	185164.954	2q32.1	Uncharacterized LOC105373782
*FSIP2*	185738.895	2q32.1	Fibrous sheath interacting protein 2
*FSIP2-AS1*	185788.020	2q32.1	FSIP2 antisense RNA 1
*LINC01473*	186033.534	2q32.1	Long intergenic non-protein coding RNA 1473
*ZC3H15*	186486.260	2q32.1	Zinc finger CCCH-type containing 15
*ITGAV*	186590.056	2q32.1	Integrin subunit alpha V
*FAM171B*	186694.060	2q32.1	Family with sequence similarity 171 member B
*ZSWIM2*	186827.480	2q32.1	Zinc finger SWIM-type containing 2
*CALCRL*	187341.965	2q32.1	Calcitonin receptor like receptor
*TFPI*	187464.231	2q32.1	Tissue factor pathway inhibitor
*LINC01090*	188035.596	2q32.1	Long intergenic non-protein coding RNA 1090
*MIR561*	188297.492	2q32.1	MicroRNA 561
*GULP1*	188291.874	2q32.1–q32.2	GULP PTB domain containing engulfment adaptor 1

Three among these genes (*MIR4437* , *MIR548AE1* , and *MIR561*) that reside within 2q31.3–q32.1 encode miRNAs. Target genes of these miRNAs were retrieved from the miRDB [25] with a cut-off for prediction score ≥95 (Supplementary Table 2). Experimental evidence for these miRNA targets that reside on chromosome 2 was retrieved from miRTarBase [26] (Supplementary Table 2). None of these target genes are located within the previously reported genomic coordinates of the causative agent of DA10.

**Fig. 3:**
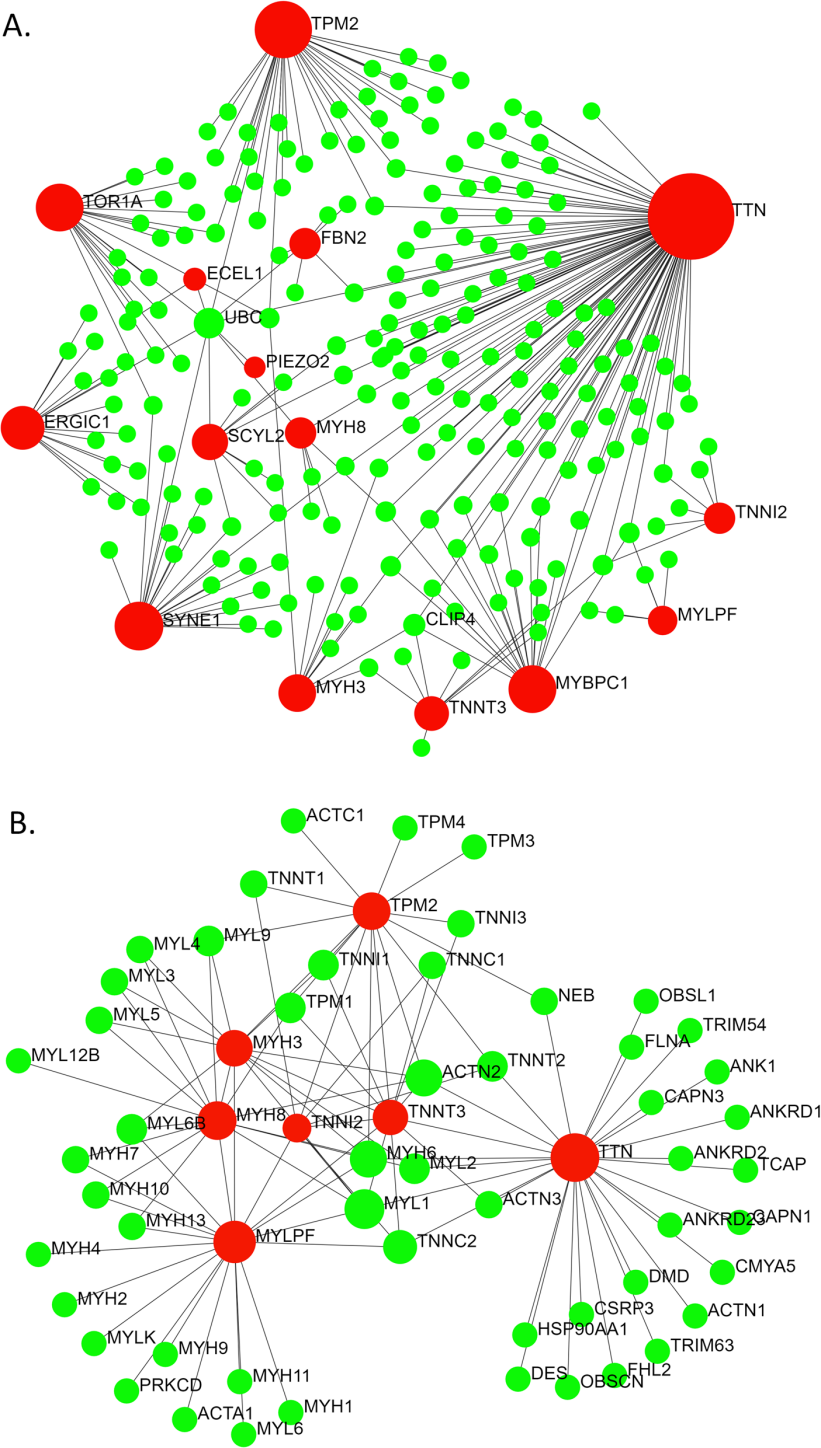
PPI networks generated using the genes along with 15 known genes associated with AMCs and DAs based on (**A**) IMEx and (**B**) STRING interactome databases through NetworkAnalyst. AMC and DA-associated gene-encoded proteins along with TTN are shown in red and the other interacting proteins are shown in green

**Fig. 4:**
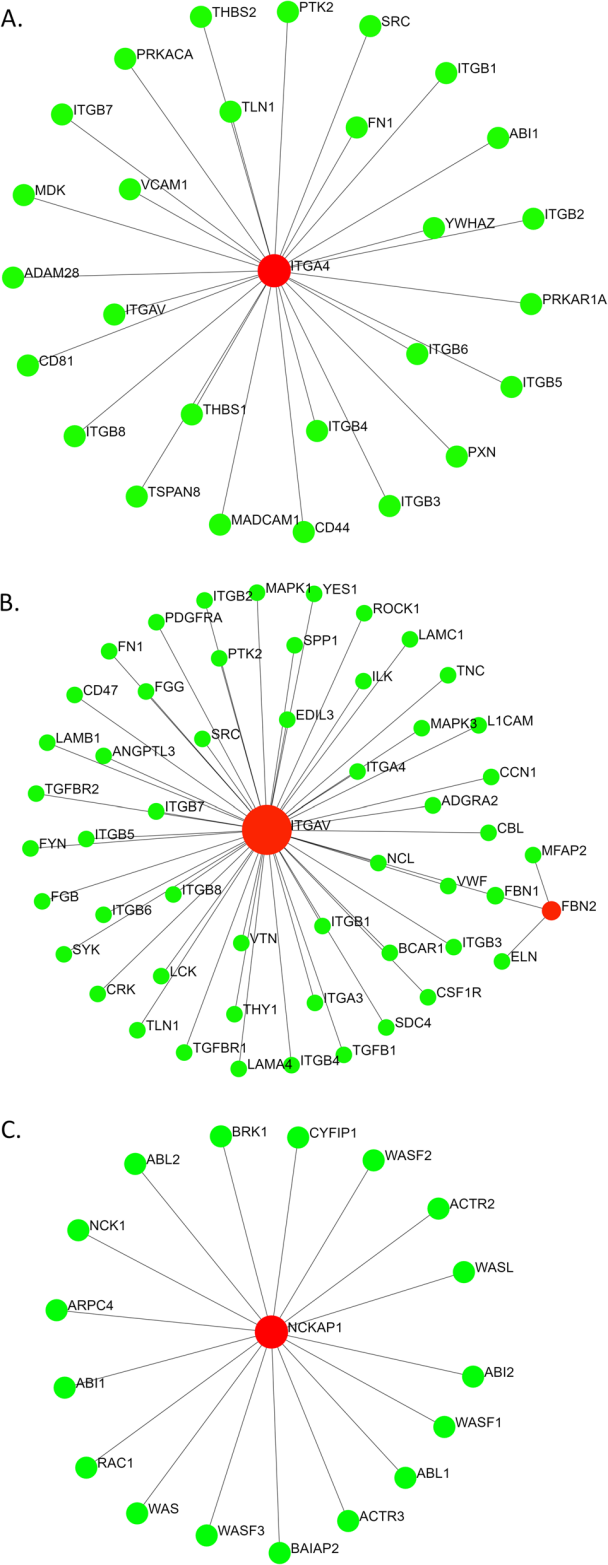
Interactions of ITGA4 (**A**), ITGAV (**B**) and NCKAP1 (**C**) with the known AMC and DA-associated proteins. Only the interactions with a high confidence score and experimental evidences are shown in the figure. AMC and DA-associated gene-encoded proteins along with ITGAV, ITGA4, and NCKAP1 are shown in red and the other interacting proteins are shown in green

The four potential candidate gene products- ITGA4, ITGAV, NCKAP1, and TTN*,* were used as inputs along with the other DA-associated genes in the STRING interactome database (with experimental evidence and high confidence score) to assess the most probable candidate gene for DA10. Input of *TTN* along with the 15 known AMCs and DAs associated genes generated a more extensive PPI network with 230 nodes (Fig. 3). ITGAV was found to be associated only with the FBN2 gene (associated with DA9) in the same network at this confidence level (Fig. 4). ITGA4 and NCKAP1 appeared to form isolated sub-networks (Fig. 4).

## Discussion

In this study, we used in silico tools and databases to identify the candidate gene for distal arthrogryposis type 10. Our exploration suggests the involvement of *Titin (TTN)* in the development of DA10.

*TTN* is a large gene with 364 exons and multiple splice variants [27]. *TTN* is expressed in the striated muscles– cardiac and skeletal muscles [28]. Since *TTN* is not expressed in other tissues, non-muscular abnormalities are not supposed to occur in titinopathies. This is consistent with the results from a previous study on DA10 patients, which found all DA10 patients to have normal neurological responses, electromyographic patterns, and creatinine kinase levels [12]. The majority of the *TTN*-related skeletal muscle disorders follow the autosomal-dominant pattern of inheritance [27], which is a common characteristic of distal arthrogryposes as well [12].

The N2A isoform of TTN is predominantly present in the skeletal muscles, and this N2A isoform excludes some of the exons of predominant cardiac isoforms N2B and N2BA. That is why, mutations of *TTN* can result in isolated cardiomyopathies, isolated skeletal muscle disorders, and combined cardiac-skeletal muscle diseases [27].

Tibial muscular dystrophy (TMD) is caused by mutations in the *TTN* and manifests itself in the adult patient as weakening of the tibialis anterior muscle [27]. In Finnish TMD patients, FINmaj mutation (an 11-bp deletion) was found in the last exon of *TTN* [29]. TMD patients having pelvic and shoulder muscle disorders were found to carry homozygous FINmaj mutations [27]. Other frameshift and missense mutations in the *TTN* contributed to phenotypic severity among the TMD patients [30]. Similarly, DA10 patients also show variability in the number and severity of affected joints [12].

There are reports on the involvement of *TTN* in congenital contractures. Fernández-Marmiesse *et al* have shown that a homozygous deletion of 5-bp in a prenatal exon of *TTN* (exon 197) causes a frameshift leading to a premature truncated protein, which results in congenital contractures [31]. Chervinsky et al. have shown another lethal contracture syndrome, where homozygous deletion is present in exon 167 of *TTN*, which is part of the fetal TTN isoform [32]. Bryen et al. have reported autosomal recessive form of arthrogryposis in patients having splice-site variant at intron 213 of *TTN* [33]. These observations clearly state that a wide range of mutations can change the characteristics of TTN for disease development, sometimes resulting in arthrogryposis. This is not uncommon, since different mutations in PIEZO2, TPM2, and MYH3 are known to cause different types of DAs.

The biological pathways and processes associated with these 151 nodal proteins (Table 2) are shared by different distal arthrogryposes. For example, patients with DA type 1 and its subtypes show mutations in genes TPM2, MYBPC1, and MYLPF that cause impairment of muscle contraction, and affect the binding of actin filament [34–36]. Patients with DA type 2 and DA type 7 have issues with muscle development because of mutations in the troponin and myosin proteins [37–40]. On the other hand, the most prominent pathways identified through the KEGG database included hypertrophic cardiomyopathy, dilated cardiomyopathy, cardiac muscle contraction, and adrenergic signaling in cardiomyocytes. Since no report has established links of hypertrophic cardiomyopathy, dilated cardiomyopathy, cardiac muscle contraction, and/or adrenergic signaling in cardiomyocytes with distal arthrogryposis, this calls for further exploration in DA patients. The genes associated with these pathways are shown in Supplementary Figure 1.

As shown in Tables 2 and 3, the nodes (proteins) in the PPI network (Fig. 1) participate in pathways like actin filament-based movement, muscle contraction, cytoskeleton organization, muscle organ development, cardiomyopathy, ITGA4, ITGAV, and NCKAP1, which are all found in the 2q31.3–q32.2 chromosomal region, where the candidate gene for DA10 is thought to lie, do not appear to be viable candidates for DA10 because they are not seen in skeletal or muscular disorders to any significant level [12]. NCKAP1 has been shown to be associated with cancer metastasis in non-small cell lung carcinoma [41], hepatocellular carcinoma [42], autism [43], and Alzheimer’s disease [44]. ITGA4 is associated with multiple sclerosis [45], autism [46], and metastasis of cholangiocarcinoma [47], among others. ITGAV is associated in many conditions as melanoma [48], prostate cancer adhesion [49], and rheumatoid arthritis [50], etc.

Tissue-specific expression profiles also reveal similarities between *TTN* and other DA-associated genes [28]. Other than *TPM2* and *PIEZO2*, gene expression profiles of all other candidate genes of different distal arthrogryposis are restricted to specific organs (Supplementary figure 2). *TTN* appears to share this characteristic with other DA-associated genes, as *TTN* is expressed only in the striated muscle tissues (Supplementary figure 2). As mentioned earlier, DA10 is distinguished from the AMCs and other DAs by plantar flexion contractures, resulting in toe-walking during infancy, in addition to variable contractures of the hip, hamstring, elbow, wrist, and finger joints [12, 13]. These symptoms indicate impairment in the musculoskeletal system. On the contrary, *NCKAP1* and *ITGAV* have generalized expression patterns in different tissues, whereas *ITGA4* expression is more restricted to cells of immune and lymphatic systems (Supplementary figure 3) [28]. *NCKAP1*, *ITGAV*, and *ITGA4* do not show specificity or high expressivity at the muscular tissues.

There are several sources that linked DA10 with the F-box protein 8 (FBXO8) [51, 52]. However, the cytogenetic location of FBXO8 is 4q34.1 [6], which contradicts with the previously identified location of DA10 candidate gene at 2q31.3–q32.2 [12]. Additionally, *FBXO8* shows a generalized expression pattern, with no expression in the skeletal, smooth, or heart muscle (Supplementary figure 4). FBXO8 protein also does not interact with the other DA-associated proteins in the PPI network. Considering these facts, we hypothesize *TTN* to be the candidate gene of DA10. Association of *TTN* with DA10 may be investigated further using targeted gene sequencing of DA10 patients. Knowledge about the genetic basis of DA10 may aid in understanding the pathogenesis mechanism as well as developing more effective therapeutic strategies in the future.

## Conclusions

In this study, we scrutinized the protein-protein interaction (PPI) networks as well as the associated biological processes and pathways to identify the candidate gene for DA10. *TTN* resides within the previously reported genomic coordinates of the potential candidate gene of DA10. *TTN* is predominantly expressed in the skeletal and heart muscles and its expression follows a pattern similar to the other known DA-associated genes. TTN participates in biological pathways and processes relevant to arthrogryposis. Based on the findings of these *in silico* analyses and their correlation with previous reports, *TTN* appears to be the candidate gene for DA10.

## Supplementary Information

**Additional file 1: Supplementary Table 1.** List of interacting proteins in the network shown in Fig. 1.

**Additional file 2: Supplementary Table 2.** List of target genes of *MIR4437*, *MIR548AE1* and *MIR561* encoded miRNAs.

**Additional file 3: Supplementary Figure 1.** The DA associated genes (in red) that participate in hypertrophic cardiomyopathy, dilated cardiomyopathy and/or adrenergic signaling in cardiomyocytes pathways. The other nodes that participate in these pathways are shown in blue. The rest of the interacting proteins are shown in green.

**Additional file 4: Supplementary Figure 2.** Tissue specific expression profiles of *TTN* and genes known to be associated with DAs [28].

**Additional file 5: Supplementary Figure 3.** Tissue specific expression profiles of *ITGA4*, *ITGAV*, and *NCKAP1* [28].

**Additional file 6: Supplementary Figure 4.** Tissue specific expression profiles of FBXO2 [28].

## Data Availability

All data generated or analyzed during this study are included in this published article (and its supplementary information files).

## Abbreviations

AMC: Arthrogryposis multiplex congenita
DA: Distal arthrogryposis
DA10: Distal arthrogryposis type 10
FDR: False discovery rate
PPI: Protein-protein interaction
