# Supplementary material for: TTN as a candidate gene for distal arthrogryposis type 10 pathogenesis
Source: J Genet Eng Biotechnol. 2022 Aug 11;20:119. doi: 10.1186/s43141-022-00405-5 (PMC9372250; doi:10.1186/s43141-022-00405-5)
Supplement: Supplementary file 1 — Additional file 1: Supplementary Table 1. List of interacting proteins in the network shown in Fig. 1. [file 43141_2022_405_MOESM1_ESM.doc]

**Supplementary table 1:** List of interacting proteins in the network shown in figure 1.

| **Gene** | **Degree/interacting nodes** |
| --- | --- |
| TPM2 | 31 |
| SYNE1 | 20 |
| MYBPC1 | 19 |
| TOR1A | 19 |
| ERGIC1 | 15 |
| MYH3 | 10 |
| SCYL2 | 9 |
| UBC | 9 |
| TNNT3 | 8 |
| FBN2 | 6 |
| MYH8 | 6 |
| TNNI2 | 6 |
| MYL1 | 5 |
| MYLPF | 5 |
| ACTN2 | 4 |
| MYH6 | 4 |
| CLIP4 | 3 |
| MYL6B | 3 |
| MYL9 | 3 |
| SNAPIN | 3 |
| TNNC2 | 3 |
| TNNI1 | 3 |
| DYNC1H1 | 2 |
| DYSF | 2 |
| ECEL1 | 2 |
| ESR1 | 2 |
| LMNA | 2 |
| MYBPC2 | 2 |
| MYH10 | 2 |
| MYH13 | 2 |
| MYH7 | 2 |
| MYL2 | 2 |
| MYL3 | 2 |
| MYL4 | 2 |
| MYL5 | 2 |
| SGCG | 2 |
| TNNI3 | 2 |
| TNNT1 | 2 |
| TNNT2 | 2 |
| TRIM63 | 2 |
| TULP3 | 2 |
| ACO1 | 1 |
| ACTA1 | 1 |
| ACTC1 | 1 |
| ACTN3 | 1 |
| AHCYL1 | 1 |
| AKT1 | 1 |
| AMOT | 1 |
| ANKRD1 | 1 |
| APC | 1 |
| APPL1 | 1 |
| ARRB1 | 1 |
| ARRB2 | 1 |
| ASH2L | 1 |
| ATP6V1B1 | 1 |
| C1QTNF9 | 1 |
| CALM1 | 1 |
| CANX | 1 |
| CAPN3 | 1 |
| CDC42 | 1 |
| CDC5L | 1 |
| CDK2 | 1 |
| CEP63 | 1 |
| CNIH4 | 1 |
| COPS4 | 1 |
| CORO1B | 1 |
| CPSF7 | 1 |
| CUL1 | 1 |
| DDB1 | 1 |
| DDX3X | 1 |
| DDX46 | 1 |
| DHCR7 | 1 |
| DISC1 | 1 |
| DNAJB5 | 1 |
| DNAJB6 | 1 |
| DSTN | 1 |
| DTNBP1 | 1 |
| EIF4A3 | 1 |
| ELAVL1 | 1 |
| ELN | 1 |
| EMD | 1 |
| EPB41 | 1 |
| ERGIC3 | 1 |
| ESRRA | 1 |
| FAF2 | 1 |
| FBXO2 | 1 |
| FBXO6 | 1 |
| FHL1 | 1 |
| GFI1 | 1 |
| GNAI2 | 1 |
| GOLGB1 | 1 |
| H2AFX | 1 |
| HAP1 | 1 |
| HECTD1 | 1 |
| HIGD1A | 1 |
| HNF4G | 1 |
| ICT1 | 1 |
| IST1 | 1 |
| ITGA2 | 1 |
| KBTBD10 | 1 |
| KIAA0101 | 1 |
| KIAA0368 | 1 |
| KLHL14 | 1 |
| LAMTOR5 | 1 |
| LIN37 | 1 |
| LRRC7 | 1 |
| LRRK2 | 1 |
| MAGOH | 1 |
| MAST2 | 1 |
| MATN2 | 1 |
| MCC | 1 |
| MEOX2 | 1 |
| MRPL11 | 1 |
| MRPL32 | 1 |
| MUSK | 1 |
| MYH1 | 1 |
| MYH11 | 1 |
| MYH2 | 1 |
| MYH4 | 1 |
| MYH9 | 1 |
| MYL12B | 1 |
| MYL6 | 1 |
| MYLK | 1 |
| MYOM1 | 1 |
| NCF1 | 1 |
| NDEL1 | 1 |
| NDUFV1 | 1 |
| NEB | 1 |
| NONO | 1 |
| NOP56 | 1 |
| NUDT3 | 1 |
| OPTN | 1 |
| PDLIM7 | 1 |
| PHB2 | 1 |
| PIEZO2 | 1 |
| PPP1R7 | 1 |
| PPP2CB | 1 |
| PPP2R4 | 1 |
| PRKCD | 1 |
| PRKD3 | 1 |
| PTEN | 1 |
| RORB | 1 |
| RRAD | 1 |
| SF3B2 | 1 |
| SH3BP5 | 1 |
| SKIL | 1 |
| SMURF2 | 1 |
| SNCA | 1 |
| SNUPN | 1 |
| SOD2 | 1 |
| SP1 | 1 |
| SPEN | 1 |
| SRXN1 | 1 |
| STAM | 1 |
| STMN1 | 1 |
| STON2 | 1 |
| SULT1A1 | 1 |
| SUN1 | 1 |
| SUN2 | 1 |
| TAF15 | 1 |
| TERF2 | 1 |
| TNIK | 1 |
| TNNC1 | 1 |
| TOMM7 | 1 |
| TOR1AIP1 | 1 |
| TOR1AIP2 | 1 |
| TOR1B | 1 |
| TOR3A | 1 |
| TP53RK | 1 |
| TP63 | 1 |
| TPM1 | 1 |
| TPM3 | 1 |
| TPM4 | 1 |
| TSG101 | 1 |
| TTN | 1 |
| UQCRFS1 | 1 |
| USP19 | 1 |
| USP25 | 1 |
| WASL | 1 |
| ZFYVE9 | 1 |
