# Supplementary material for: TTN as a candidate gene for distal arthrogryposis type 10 pathogenesis
Source: J Genet Eng Biotechnol. 2022 Aug 11;20:119. doi: 10.1186/s43141-022-00405-5 (PMC9372250; doi:10.1186/s43141-022-00405-5)
Supplement: Supplementary file 2 — Additional file 2: Supplementary Table 2. List of target genes of MIR4437, MIR548AE1 and MIR561 encoded miRNAs. [file 43141_2022_405_MOESM2_ESM.doc]

**Supplementary table 2:** List of target genes of [*MIR4437*](http://atlasgeneticsoncology.org/Genes/GC_MIR4437.html), [*MIR548AE1*](http://atlasgeneticsoncology.org/Genes/GC_MIR548AE1.html) and [*MIR561*](http://atlasgeneticsoncology.org/Genes/GC_MIR561.html) encoded miRNAs.

| **Target Gene Symbol*** | **miRNA** | **Chromosomal location** | **Target Score** | **Experimental evidence^#^** |
| --- | --- | --- | --- | --- |
| *B3GALT2* | *MIR548AE1* | 1: 193178730-193186613 | 100 | **-** |
| *PRMT3* | *MIR548AE1* | 11: 20387716-20509338 | 100 | **-** |
| *DYRK2* | *MIR548AE1* | 12: 67648745-67665406 | 100 | **-** |
| *GPC6* | *MIR548AE1* | 13: 93216529-94408020 | 100 | **-** |
| *SOS2* | *MIR548AE1* | 14: 50117130-50231882 | 100 | **-** |
| *ARIH1* | *MIR561* | 15: 72474330-72602987 | 100 | **-** |
| *CPEB1* | *MIR548AE1* | 15: 82543201-82648795 | 100 | **-** |
| *PRSS8* | *MIR4437* | 16: 31131433-31135727 | 100 | **-** |
| *GNG13* | *MIR4437* | 16: 798041-800734 | 100 | **-** |
| *DNMT1* | *MIR548AE1* | 19: 10133346-10194953 | 100 | **-** |
| ***IKZF2*** | ***MIR548AE1*** | **2: 212999698-213152456** | **100** | **None** |
| *TNRC6B* | *MIR548AE1* | 22: 40044834-40335808 | 100 | **-** |
| *PHC3* | *MIR548AE1* | 3: 170087584-170181733 | 100 | **-** |
| *RBPJ* | *MIR548AE1* | 4: 26105449-26435131 | 100 | **-** |
| *CREBRF* | *MIR548AE1* | 5: 173056352-173139284 | 100 | **-** |
| *CPEB4* | *MIR548AE1* | 5: 173888349-173961980 | 100 | **-** |
| *PTPRK* | *MIR548AE1* | 6: 127968785-128520599 | 100 | **-** |
| *MED14* | *MIR561* | X: 40648305-40736159 | 100 | **-** |
| *KDM5B* | *MIR548AE1* | 1: 202724495-202808421 | 99 | **-** |
| *ACBD3* | *MIR561* | 1: 226144679-226186741 | 99 | **-** |
| *HNRNPR* | *MIR548AE1* | 1: 23304688-23344284 | 99 | **-** |
| *B3GALNT2* | *MIR548AE1* | 1: 235439796-235504452 | 99 | **-** |
| *TUT4* | *MIR548AE1* | 1: 52423275-52553463 | 99 | **-** |
| *ARHGAP29* | *MIR548AE1* | 1: 94168905-94314592 | 99 | **-** |
| *PTBP2* | *MIR548AE1* | 1: 96721784-96823739 | 99 | **-** |
| *CELF2* | *MIR548AE1* | 10: 10462550-11336675 | 99 | **-** |
| *HECTD2* | *MIR548AE1* | 10: 91409235-91514820 | 99 | **-** |
| *CNTN1* | *MIR561* | 12: 40692439-41072415 | 99 | **-** |
| *KCTD12* | *MIR548AE1* | 13: 76880175-76886405 | 99 | **-** |
| *STRN3* | *MIR548AE1* | 14: 30893804-31026379 | 99 | **-** |
| *ARID4A* | *MIR548AE1* | 14: 58298555-58373876 | 99 | **-** |
| *PCNX1* | *MIR561* | 14: 70907459-71115382 | 99 | **-** |
| *GTF2A1* | *MIR548AE1* | 14: 81175452-81221390 | 99 | **-** |
| *TJP1* | *MIR561* | 15: 29699367-29969049 | 99 | **-** |
| *SLC12A1* | *MIR561* | 15: 48206302-48304078 | 99 | **-** |
| *MAPK6* | *MIR548AE1* | 15: 51971825-52067375 | 99 | **-** |
| *C16orf72* | *MIR561* | 16: 9091644-9121635 | 99 | **-** |
| *TVP23B* | *MIR548AE1* | 17: 18781183-18806714 | 99 | **-** |
| *MED13* | *MIR548AE1* | 17: 61942605-62065278 | 99 | **-** |
| *DDA1* | *MIR548AE1* | 19: 17309563-17323298 | 99 | **-** |
| *ELAVL1* | *MIR548AE1* | 19: 7958573-8005641 | 99 | **-** |
| ***ZEB2*** | ***MIR561*** | **2: 144384081-144520119** | **99** | **None** |
| ***ACVR2A*** | ***MIR561*** | **2: 147844517-147930822** | **99** | **NGS, CLIP-Seq** |
| ***CCDC85A*** | ***MIR561*** | **2: 56183852-56386174** | **99** | **None** |
| ***ACTR2*** | ***MIR561*** | **2: 65227831-65271253** | **99** | **CLIP-Seq** |
| ***GFPT1*** | ***MIR548AE1*** | **2: 69319780-69387227** | **99** | **None** |
| *SKIL* | *MIR548AE1* | 3: 170357715-170396849 | 99 | **-** |
| *RBMS3* | *MIR548AE1* | 3: 29281071-30010395 | 99 | **-** |
| *DAG1* | *MIR548AE1* | 3: 49468948-49535615 | 99 | **-** |
| *ROBO1* | *MIR561* | 3: 78597239-79767998 | 99 | **-** |
| *CADM2* | *MIR548AE1* | 3: 84958989-86074429 | 99 | **-** |
| *VGLL3* | *MIR548AE1* | 3: 86937973-86991149 | 99 | **-** |
| *TET2* | *MIR548AE1* | 4: 105145875-105279803 | 99 | **-** |
| *SLC4A4* | *MIR561* | 4: 71062660-71572083 | 99 | **-** |
| *APC* | *MIR561* | 5: 112707498-112846239 | 99 | **-** |
| *RBM27* | *MIR548AE1* | 5: 146203605-146289223 | 99 | **-** |
| *RETREG1* | *MIR561* | 5: 16473053-16616997 | 99 | **-** |
| *BDP1* | *MIR548AE1* | 5: 71455651-71578288 | 99 | **-** |
| *IQGAP2* | *MIR548AE1* | 5: 76403285-76708132 | 99 | **-** |
| *EDIL3* | *MIR561* | 5: 83940554-84384880 | 99 | **-** |
| *TMEM181* | *MIR561* | 6: 158536640-158635429 | 99 | **-** |
| *SYNCRIP* | *MIR548AE1* | 6: 85607784-85643870 | 99 | **-** |
| *MYC* | *MIR548AE1* | 8: 127735434-127742951 | 99 | **-** |
| *FZD3* | *MIR548AE1* | 8: 28494212-28574258 | 99 | **-** |
| *RPS6KA3* | *MIR548AE1* | X: 20149911-20267097 | 99 | **-** |
| *RSBN1* | *MIR548AE1* | 1: 113761832-113812476 | 98 | **-** |
| *PTGFRN* | *MIR4437* | 1: 116909916-116990353 | 98 | **-** |
| *RC3H1* | *MIR548AE1* | 1: 173931084-174022357 | 98 | **-** |
| *NEK7* | *MIR561* | 1: 198156998-198322420 | 98 | **-** |
| *PRKAA2* | *MIR548AE1* | 1: 56645314-56715335 | 98 | **-** |
| *ZNF326* | *MIR548AE1* | 1: 89995110-90035533 | 98 | **-** |
| *ZC3H12C* | *MIR561* | 11: 110092506-110171841 | 98 | **-** |
| *ZC3H12C* | *MIR548AE1* | 11: 110092506-110171841 | 98 | **-** |
| *SOX6* | *MIR548AE1* | 11: 15966449-16738477 | 98 | **-** |
| *ATF7IP* | *MIR561* | 12: 14365682-14502930 | 98 | **-** |
| *ETNK1* | *MIR548AE1* | 12: 22625171-22690665 | 98 | **-** |
| *TM7SF3* | *MIR548AE1* | 12: 26971579-27014384 | 98 | **-** |
| *PAN3* | *MIR548AE1* | 13: 28138193-28295335 | 98 | **-** |
| *PDS5B* | *MIR548AE1* | 13: 32586452-32778019 | 98 | **-** |
| *GTF2A1* | *MIR561* | 14: 81175452-81221390 | 98 | **-** |
| *RORA* | *MIR548AE1* | 15: 60488284-61229302 | 98 | **-** |
| *TVP23C* | *MIR548AE1* | 17: 15502264-15563483 | 98 | **-** |
| *APPBP2* | *MIR548AE1* | 17: 60443158-60526242 | 98 | **-** |
| *KLHL14* | *MIR561* | 18: 32672673-32773023 | 98 | **-** |
| *EPG5* | *MIR548AE1* | 18: 45800581-45967329 | 98 | **-** |
| *SOCS6* | *MIR548AE1* | 18: 70289045-70330199 | 98 | **-** |
| ***MAP3K2*** | ***MIR548AE1*** | **2: 127298668-127388465** | **98** | **None** |
| ***IWS1*** | ***MIR561*** | **2: 127480812-127527336** | **98** | **None** |
| ***NAB1*** | ***MIR548AE1*** | **2: 190648895-190692766** | **98** | **None** |
| ***KANSL1L*** | ***MIR548AE1*** | **2: 210021421-210172760** | **98** | **None** |
| *CEBPB* | *MIR561* | 20: 50190583-50192690 | 98 | **-** |
| *B4GALT4* | *MIR548AE1* | 3: 119211742-119240878 | 98 | **-** |
| *ZNF148* | *MIR548AE1* | 3: 125225669-125375354 | 98 | **-** |
| *KPNA4* | *MIR548AE1* | 3: 160495007-160565571 | 98 | **-** |
| *ACVR2B* | *MIR548AE1* | 3: 38453890-38493142 | 98 | **-** |
| *ROBO1* | *MIR548AE1* | 3: 78597239-79767998 | 98 | **-** |
| *DCBLD2* | *MIR548AE1* | 3: 98795941-98901695 | 98 | **-** |
| *FHDC1* | *MIR548AE1* | 4: 152911339-152979671 | 98 | **-** |
| *ECT2L* | *MIR561* | 6: 138796087-138904070 | 98 | **-** |
| *FAM135A* | *MIR561* | 6: 70413508-70561174 | 98 | **-** |
| *HERPUD2* | *MIR548AE1* | 7: 35632659-35695135 | 98 | **-** |

*Only the target genes that reside on chromosome 2 are shown as bold.

^#^ Experimental evidences only for the target genes that reside on chromosome 2 are shown.
