# Supplementary figures and images for: TTN as a candidate gene for distal arthrogryposis type 10 pathogenesis
Source: J Genet Eng Biotechnol. 2022 Aug 11;20:119. doi: 10.1186/s43141-022-00405-5 (PMC9372250; doi:10.1186/s43141-022-00405-5)

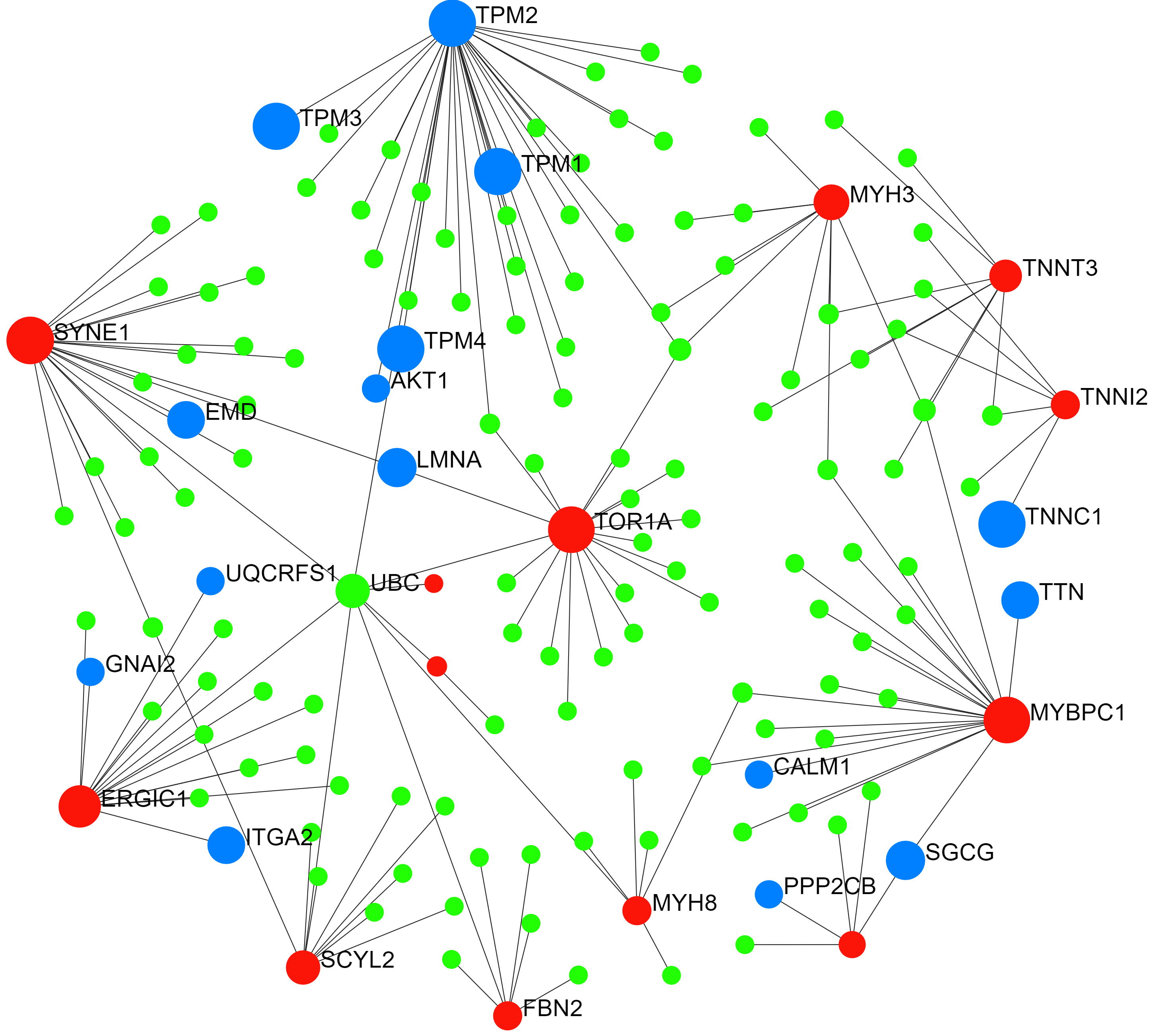

Supplement: Supplementary file 3 — Additional file 3: Supplementary Figure 1. The DA associated genes (in red) that participate in hypertrophic cardiomyopathy, dilated cardiomyopathy and/or adrenergic signaling in cardiomyocytes pathways. The other nodes that participate in these pathways are shown in blue. The rest of the interacting proteins are shown in green. [file 43141_2022_405_MOESM3_ESM.tif]

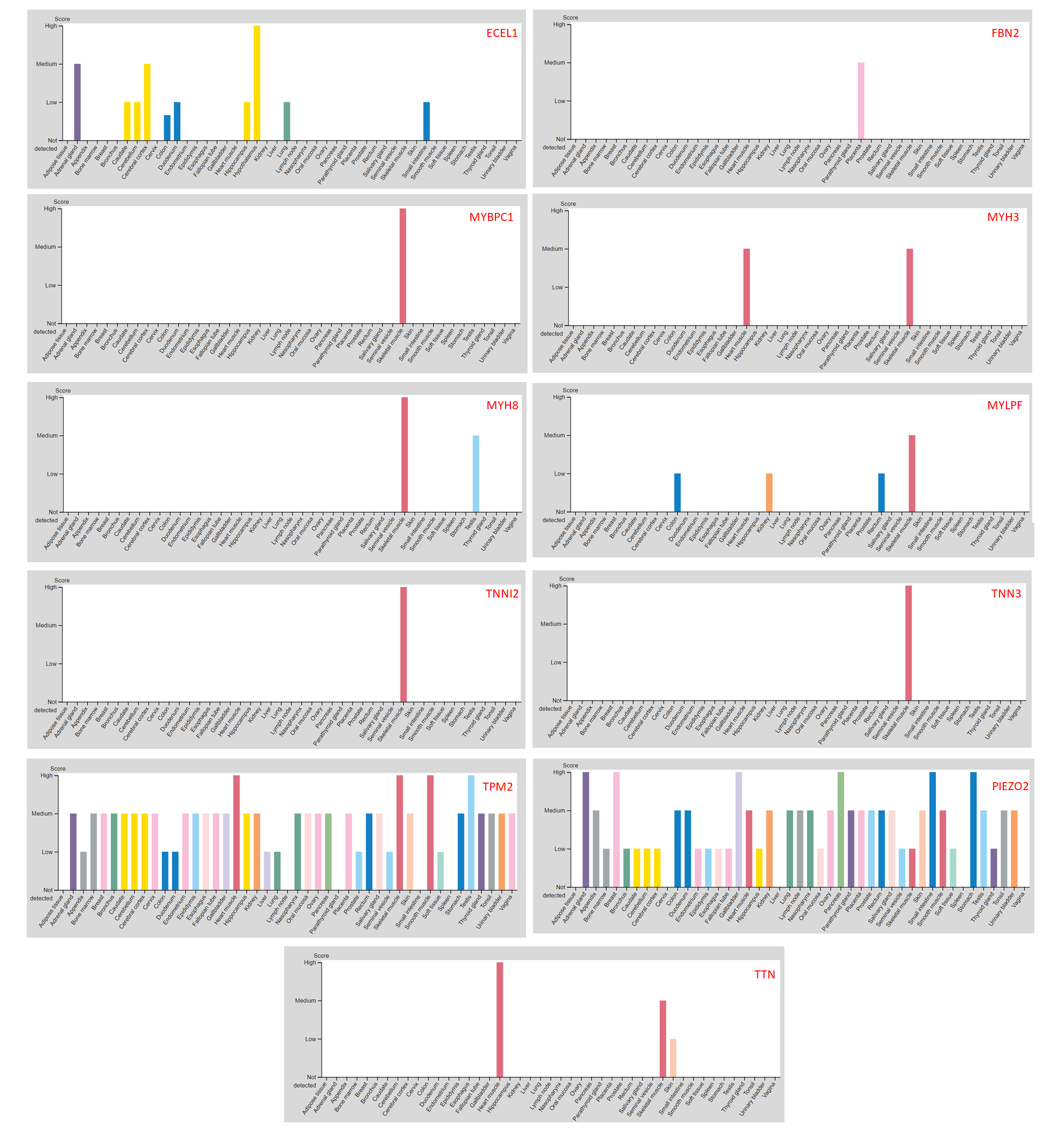

Supplement: Supplementary file 4 — Additional file 4: Supplementary Figure 2. Tissue specific expression profiles of TTN and genes known to be associated with DAs [28]. [file 43141_2022_405_MOESM4_ESM.tif]

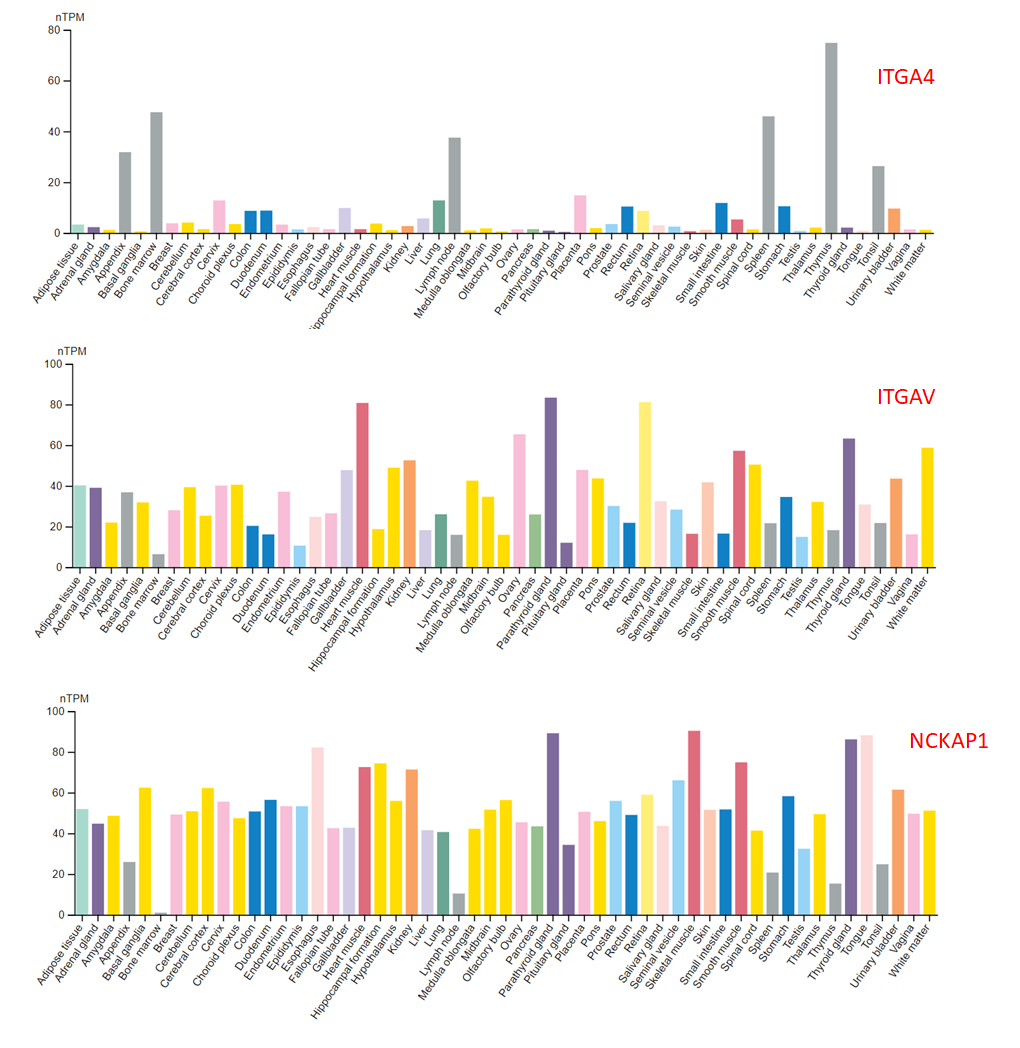

Supplement: Supplementary file 5 — Additional file 5: Supplementary Figure 3. Tissue specific expression profiles of ITGA4, ITGAV, and NCKAP1 [28]. [file 43141_2022_405_MOESM5_ESM.tif]

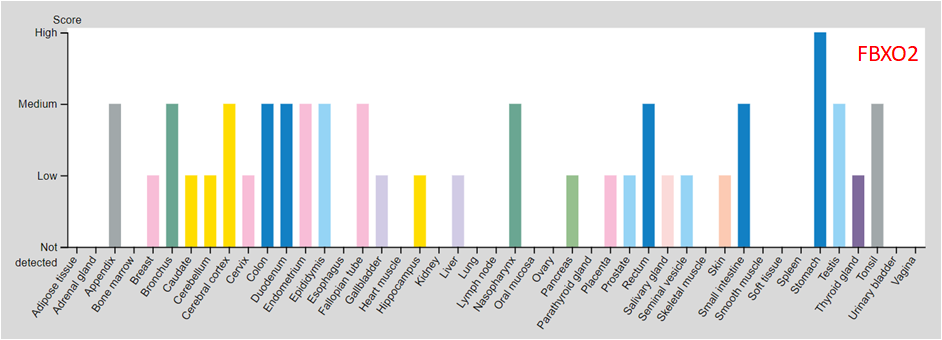

Supplement: Supplementary file 6 — Additional file 6: Supplementary Figure 4. Tissue specific expression profiles of FBXO2 [28]. [file 43141_2022_405_MOESM6_ESM.tif]
